# Tuberculosis Screening by Tuberculosis Skin Test or QuantiFERON®-TB Gold In-Tube Assay among an Immigrant Population with a High Prevalence of Tuberculosis and BCG Vaccination

**DOI:** 10.1371/journal.pone.0082727

**Published:** 2013-12-19

**Authors:** John A. Painter, Edward A. Graviss, Hoang Hoa Hai, Duong Thi Cam Nhung, Tran Thi Thanh Nga, Ngan P. Ha, Kirsten Wall, Le Thien Huong Loan, Matt Parker, Lilia Manangan, Rick O’Brien, Susan A. Maloney, R. M. Hoekstra, Randall Reves

**Affiliations:** 1 National Center for Emerging and Zoonotic Infectious Diseases, Centers for Disease Control and Prevention, Atlanta, Georgia, United States of America; 2 The Methodist Hospital Research Institute, Houston, Texas, United States of America; 3 Cho Ray Hospital, Visa Medical Unit, Ho Chi Minh City, Vietnam; 4 Denver Health and Hospital Authority, Denver, Colorado, United States of America; 5 National Center for HIV, Hepatitis, STD, and TB Prevention, Centers for Disease Control and Prevention, Atlanta, Georgia, United States of America; 6 Foundation for Innovative New Diagnostics, Geneva, Switzerland; 7 Center for Global Health, Centers for Disease Control and Prevention, Atlanta, Georgia, United States of America; 8 Biostatistics and Information Management Office, Division of Foodborne, Waterborne, and Environmental Diseases, Centers for Disease Control and Prevention, Atlanta, Georgia, United States of America; Hopital Raymond Poincare - Universite Versailles St. Quentin, France

## Abstract

**Rationale:**

Each year 1 million persons acquire permanent U.S. residency visas after tuberculosis (TB) screening. Most applicants undergo a 2-stage screening with tuberculin skin test (TST) followed by CXR only if TST-positive at > 5 mm. Due to cross reaction with bacillus Calmette-Guérin (BCG), TST may yield false positive results in BCG-vaccinated persons. Interferon gamma release assays exclude antigens found in BCG. In Vietnam, like most high TB-prevalence countries, there is universal BCG vaccination at birth.

**Objectives:**

1. Compare the sensitivity of QuantiFERON ®-TB Gold In-Tube Assay (QFT) and TST for culture-positive pulmonary TB. 2. Compare the age-specific and overall prevalence of positive TST and QFT among applicants with normal and abnormal CXR.

**Methods:**

We obtained TST and QFT results on 996 applicants with abnormal CXR, of whom 132 had TB, and 479 with normal CXR.

**Results:**

The sensitivity for tuberculosis was 86.4% for QFT; 89.4%, 81.1%, and 52.3% for TST at 5, 10, and 15 mm. The estimated prevalence of positive results at age 15–19 years was 22% and 42% for QFT and TST at 10 mm, respectively. The prevalence increased thereafter by 0.7% year of age for TST and 2.1% for QFT, the latter being more consistent with the increase in TB among applicants.

**Conclusions:**

During 2-stage screening, QFT is as sensitive as TST in detecting TB with fewer requiring CXR and being diagnosed with LTBI. These data support the use of QFT over TST in this population.

## Introduction

Foreign-born persons accounted for 60.5% of the 11,181 cases reported in 2010[1]. 

In 2010, the United States granted one million visas to permanent U.S. residents (immigrants) and three million visas to temporary workers and students (nonimmigrants)[2,3]. Of those, 45% of immigrants and 19% of nonimmigrants were from tuberculosis high-prevalence countries[4], Tuberculosis elimination in the U.S. will require detection and treatment of both tuberculosis disease and latent TB infection before or after arriving in the United States[5-7]. 

CDC mandates screening of immigrant applicants with the primary goal of detecting and treating those with infectious tuberculosis and a secondary goal of preventing future tuberculosis cases through treatment of latent tuberculosis infection[8]. For visas obtained from outside the United States, the procedure requires all applicants >14 years of age to undergo universal chest radiography, and those with findings consistent with tuberculosis must complete treatment if tuberculosis is confirmed by at least one of three required sputum specimens examined by smear for acid-fast bacilli (AFB) or mycobacterial culture[9]. For visas applicants who are residing in the United States on a temporary visa, tuberculosis screening is conducted by different two-stage process beginning with a test for tuberculosis infection followed by chest radiography only among applicants with Mantoux tuberculin skin test (TST) indurations > 5 mm in diameter or a positive interferon-gamma release assay (IGRA)[10]. For students, temporary workers, and other nonimmigrants, no tuberculosis screening is required for entry to the United States, but a two-stage process for tuberculosis screening is recommended[11-13]. Although most immigrants undergo screening for tuberculosis by this two-stage process, the outcome and the effectiveness of this approach has been evaluated only in one small study, and never compared to universal chest radiography[14]. 

Due, in part, to cross-reaction of TST with the bacillus Calmette-Guérin (BCG) vaccine, interpreting TST results and managing the risk of reactivation of LTBI in patients from high-prevalence countries are problematic[15-17]. In 2000, CDC recommended disregarding prior BCG immunization when interpreting a positive TST[12], but following the approval by the U.S. Food and Drug Administration of two interferon gamma release assays (IGRA) the recommendation were revised in 2010 to state that IGRAs were the preferred test for LTBI in populations likely to have received BCG vaccine[13]. The prevalence of the diagnosis of latent tuberculosis infection is much lower using IGRA compared to TST in many BCG-vaccinated populations that are at highest risk for TB, raising questions about the possibility of lower sensitivity of IGRA[13,18,19]. 

We compared the performance of the TST to an IGRA, QuantiFERON-TB Gold In-Tube test (QFT) among adult U.S. visa applicants undergoing radiographic screening in Vietnam, a country with universal infant BCG vaccination[20] and a tuberculosis prevalence of 334 per 100,000 population[21]. The goals of the study were to measure the sensitivity of TST and QFT in detecting culture-confirmed pulmonary tuberculosis, and to estimate the overall and age-specific prevalence of LTBI for using TST and QFT in the same adult immigrant population. 

## Methods

Institutional Review Board approval was obtained from the Centers for Disease Control and Prevention, the Cho Ray Hospital, The Methodist Hospital Research Institute, and the Denver Health and Hospital Authority. All adults provided signed consent; a parent or guardian provided signed consent on behalf of their child ages 2-17; and adolescents 15-17 signed an adolescent assent form which they will sign themselves after parental consent was obtained. Study participants were recruited from December 2008 through January 2010 from Vietnamese visa applicants during the standard immigrant medical examination at the Cho Ray Hospital Medical Visa Unit using the technical instructions published by CDC[9]. 

 Following the results of chest radiograph applicants were invited to participate in a study of TST and QFT for which they would be provided the results, but the result of which would not affect their visa application. Recruitment was done following the posterior-anterior radiograph of applicants age >14 years. First, we attempted to enroll 1,000 applicants with radiographic findings consistent with tuberculosis disease with the goal of including up to 150 with at least one sputum culture positive for Mycobacterium tuberculosis for the analysis of sensitivity[22]. Second, we also sought to enroll 500 applicants with normal chest radiographs to provide a sample large enough to calculate age-specific prevalence rates of LTBI test results. Until enrollment was completed, every applicant with a chest radiograph consistent with tuberculosis was approached for enrollment. Each week, the first available participants with a normal chest radiograph were enrolled to maintain the 2:1 ratio. 

QFT was performed on the day of enrollment, followed by TST; participants were instructed to return for TST reading in 48 to 72 hours. QFT was administered only once per participant. Purified protein derivative was purchased commercially (5 tuberculin units per 0.1mL, Pasteur institute, Nha Trang, Vietnam). QFT kits were provided by the Foundation for Innovative New Diagnostics. Prior to and at intervals during the study, we conducted onsite training and quality assurance for TST and QFT testing. We used the following materials for processing QFT samples: Biorad 550 plate reader; Biorad PW42 plate washer; Hermle Z513 centrifuge; QFT software version 2.17 (Cellestis); and QFT Kit lots 50511, 50361, and 50441. Pipette calibration was performed annually by a certified vendor. The Cho Ray technician and study quality control person (Ngan) ran QFT assays simultaneously on 20% of every fifth sample during field visits, every 6 months; of the 4-5 samples selected for quality control per visit, all results of quality control sample matched the results of the original sample. TST and QFT laboratory technicians were blinded to clinical results (i.e. chest radiograph, sputum smear and culture).

Culture was performed with both solid (Löwenstein-Jensen) and liquid (Mycobacterial Growth Indicator Tube) media (Becton, Dickinson and Company, Franklin Lakes, New Jersey). Sputum specimens were decontaminated as described by Kent and Kubica[23], with one important modification. Due to the high concentration of *Pseudomonas aeruginosa*, fungi, and mycobacteria other than tuberculosis found in Vietnam, the digesting protocol was modified from 2.0% to 2.5% NaOH[24]. *Mycobacterium tuberculosis* growth on either media was confirmed by the Gen-Probe (San Diego, USA) Accuprobes assay and conventional biochemical techniques when necessary[23,25,26]. 

The participants who were enrolled based on chest radiograph were reclassified after the results of sputum cultures into three groups as 1) having a chest radiograph not consistent with TB (Normal-CXR), 2) having a chest radiograph consistent with TB but not culture confirmed (TB-CXR), or 3) having culture-confirmed pulmonary tuberculosis (TB) when *M. tuberculosis* was isolated from any of the three sputum samples. 

To estimate the overall and age-specific prevalence of LTBI using TST or QFT, we compared the results of QFT with TST having induration >5 mm (TST-5), >10 mm (TST-10), and >15 mm (TST-15) in each participant group. To measure the sensitivity for culture-confirmed pulmonary tuberculosis, we calculated the percent positive results only among those having culture-confirmed pulmonary tuberculosis (TB). For regression analyses, ages were grouped into 5-year strata beginning at 15 years of age through age 64, and all ages 65 years or older. To estimate the expected percentage testing positive in the entire visa applicant population, we obtained the age-specific tuberculosis status of the entire applicant population during enrollment. We then weighted the study data as a random selection without replacement for those classified as Normal-CXR, TB-CXR, and TB, and summed for each 5-year age group. 

We estimated the annual percent change for having a chest radiograph consistent with tuberculosis, culture confirmed tuberculosis, and a positive TST or QFT. The annual percent change was calculated as (e^β^-1)*100, where β was the slope derived from a generalized linear model with a natural log link of individuals aggregated into 5-year age groups. 

Statistical tests were performed in R[27], version 2.14.1, by using the survey package (version 3.28) for the population estimate and the ggplot2 package (version 0.9.0) for graphs[28-30]. 

## Results

### Description of the applicant population and study participants

**Figure 1:**
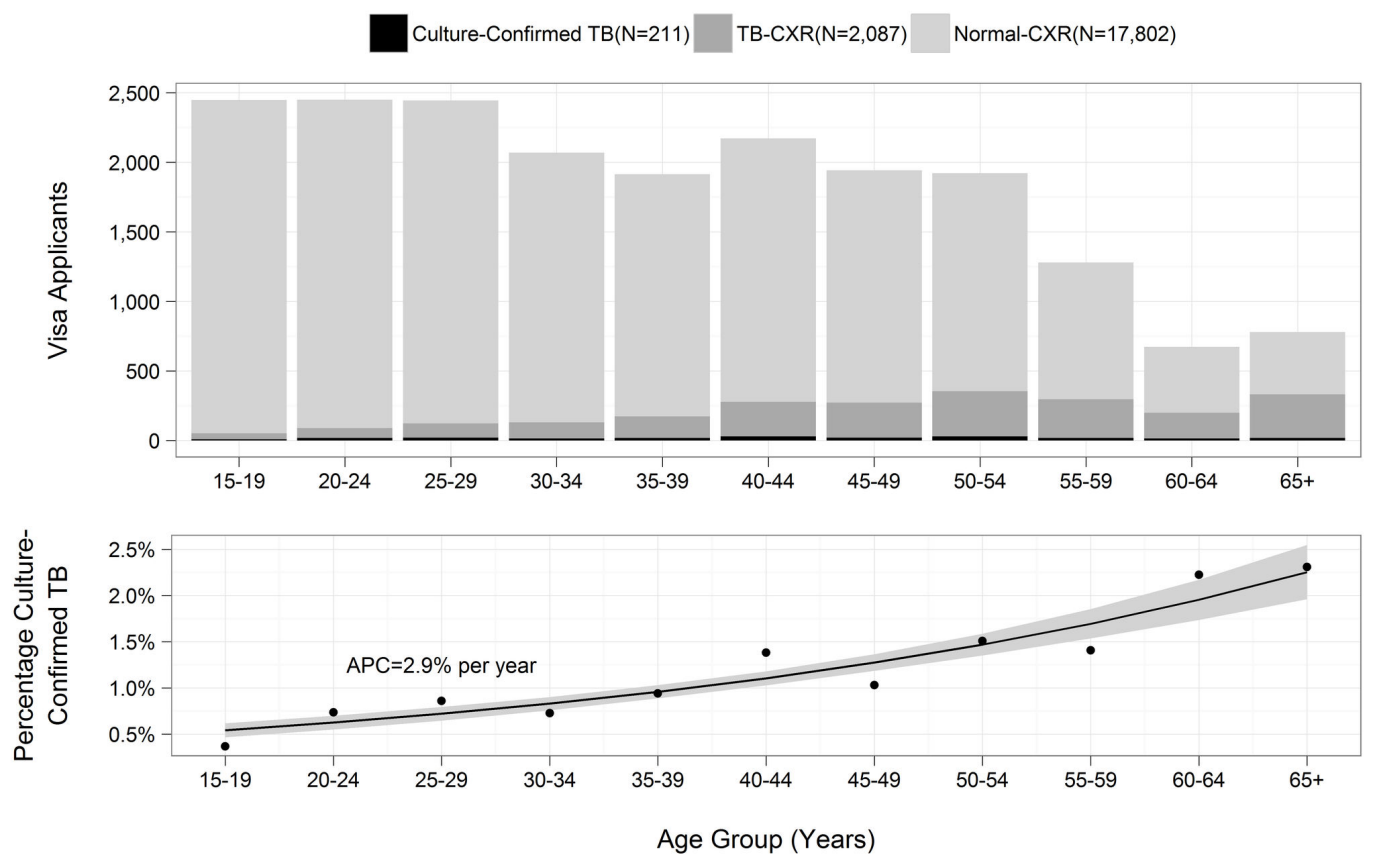
Tuberculosis screening among visa applicants. a) Number of Vietnamese applicants (N=20,100) for U.S. immigration having a chest radiograph not consistent with TB (Normal-CXR, n=17,802), having a chest radiograph consistent with TB, but not culture confirmed (TB-CXR, n=2,087), or having culture-confirmed pulmonary TB (TB, n=211) by 5-year age group. b) The percentage with a chest radiograph consistent with TB. *APC=annual percent change per year of age.

We obtained the tuberculosis classifications of the population of 20,100 visa applicants 15 years of age and older who completed the visa medical exam during the study period (Figure 1). The mean age was 37.3 years; 17,802 (88.6%) had Normal-CXR, 2,087 (10.4%) had TB-CXR, and 211 (1,040 per 100,000 population) had culture-confirmed pulmonary tuberculosis. The age-specific prevalence of tuberculosis increased with age. The annual percentage increase per year of age was 5.5% [95% confidence interval = 5.2%—5.8%] for a chest radiograph consistent with TB and 2.9% [2.0%—3.8%] for culture-confirmed TB (Figure 1b). 

We enrolled 1,475 participants 15 years of age or older of whom 479 had Normal-CXR and 996 had a chest radiograph consistent with tuberculosis (Table 1); 100 applicants declined, and 5 did not complete their examination. Of those with an abnormal CXR, 132 (13.3%) were culture-confirmed for tuberculosis (TB) and 864 were not culture confirmed (TB-CXR). Compared with study participants in the Normal-CXR group (median age=38.0 [95% exact confidence interval=37.5—39.0] years, those with an abnormal CXR (TB-CXR and TB groups combined) were more likely to be older (median age 49.5 [48.5-50.5] and 42.0 [40.0-45.5], respectively), and male (59.0% versus 48.2% [Fisher’s exact probability < 0.001]) and to self-report diabetes (3.1% versus 0.8% [Fisher’s exact probability = 0.009]), and previous tuberculosis (28.3% versus 0.2% [Fisher’s exact probability < 0.001]). Universal testing for HIV was required of U.S-immigrant applicants during the time of the study, but only one participant with HIV infection was identified among the TB group. No participants reported symptoms of tuberculosis. 

**Table 1:** Demographic and clinical characteristics of participants at least 15 years of age (N=1,475); percentage positive by QFT-TB Gold In-Tube (QFT), percentage positive by TST at indurations of 5 mm (TST-5), 10 mm (TST-10), and 15 mm (TST-15) and Mantoux tuberculin skin test response (TST), by clinical characteristic.

	Chest Radiograph Normal orNot Consistent with TB Disease										Chest Radiograph Consistent with TB Disease																
	No Culture Done (Normal-CXR)										Sputum Culture Negative (TB-CXR)									Sputum Culture Positive (TB)							
	Enrolled				% Test Positive						Enrolled			% Test Positive						Enrolled			% Test Positive				
					TST									TST									TST				
	N	(%)			5mm	10mm	15mm	QFT			N	(%)		5mm	10mm	15mm	QFT			N	(%)		5mm	10mm	15mm	QFT	
Total	479	(100)			71.0	49.3	23.0	33.6			864	(100)		79.4	61.5	33.0	67.6			132	(100)		89.4	81.1	52.3	86.4	
Age (years)	78	(16.3)			53.8	37.2	16.7	19.2			86	(10.0)		81.4	60.5	29.1	61.6			29	(22.0)		96.6	96.6	69.0	89.7	
15-29																											
30-44	311	(64.9)			74.9	51.8	23.5	36.3			206	(23.8)		88.3	72.8	44.2	72.8			43	(32.6)		88.4	81.4	53.5	86.0	
45-59	82	(17.1)			72.0	51.2	29.3	35.4			390	(45.1)		80.3	63.1	31.8	72.3			41	(31.1)		87.8	75.6	48.8	85.4	
60+	8	(1.7)			75.0	50.0	0.0	50.0			182	(21.1)		66.5	45.6	24.7	54.4			19	(14.4)		84.2	68.4	31.6	84.2	
Sex	248	(51.8)			64.9	43.5	20.6	28.2			367	(42.5)		78.7	62.4	33.0	68.4			41	(31.1)		97.6	95.1	61.0	90.2	
Female																											
Male	231	(48.2)			77.5	55.4	25.5	39.4			497	(57.5)		79.9	60.8	33.0	67.0			91	(68.9)		85.7	74.7	48.4	84.6	
History of diabetes	475	(99.2)			71.2	49.3	22.9	33.7			838	(97.0)		79.8	61.5	32.9	68.1			128	(97.0)		89.8	81.3	52.3	86.7	
No																											
Yes	4	(0.8)			50.0	50.0	25.0	25.0			26	(3.0)		65.4	61.5	34.6	50.0			4	(3.0)		75.0	75.0	50.0	75.0	
Tobacco Use	40	(8.4)			90.0	60.0	25.0	47.5			85	(9.8)		84.7	61.2	36.5	72.9			20	(15.2)		80.0	75.0	40.0	85.0	
Current																											
History only	20	(4.2)			95.0	70.0	40.0	55.0			55	(6.4)		69.1	52.7	27.3	61.8			7	(5.3)		100	85.7	57.1	100	
None	419	(87.5)			68.0	47.3	22.0	31.3			724	(83.8)		79.6	62.2	33.0	67.4			105	(79.5)		90.5	81.9	54.3	85.7	
History of TB	478	(99.8)			70.9	49.4	23.0	33.7			596	(69.0)		76.8	59.1	31.4	63.6			118	(89.4)		90.7	82.2	51.7	86.4	
No																											
Treated	1	(0.2)			100	0.0	0.0	0.0			258	(29.9)		84.9	67.1	37.2	77.1			12	(9.1)		75.0	66.7	50.0	83.3	
Not treated	.	.	.		.	.	.	.		.	10	(1.2)		90.0	60.0	20.0	60.0			2	(1.5)		100	100	100	100	

Culture-confirmed cases were identified on the first sputum sample for 95 (72.0%); 27 (20.4%) additional cases were identified on the second sputum sample; and 10 (7.6%) on the third sputum sample. One or more sputum specimens were culture-positive for non-tuberculous mycobacteria (NTM) in 6 (4.5%) of the TB group and 105 (12.2%) of the TB-CXR group. Of the 111 patients with at least one culture yielding NTM, 82 (73.9%) had only one culture yielding NTM, and only two had more than one NTM culture had at least one positive AFB smear. These findings are most consistent with low-level contamination for NTM rather than lung disease due to NTM. 

### Sensitivity of TST and QFT for TB

**Figure 2:**
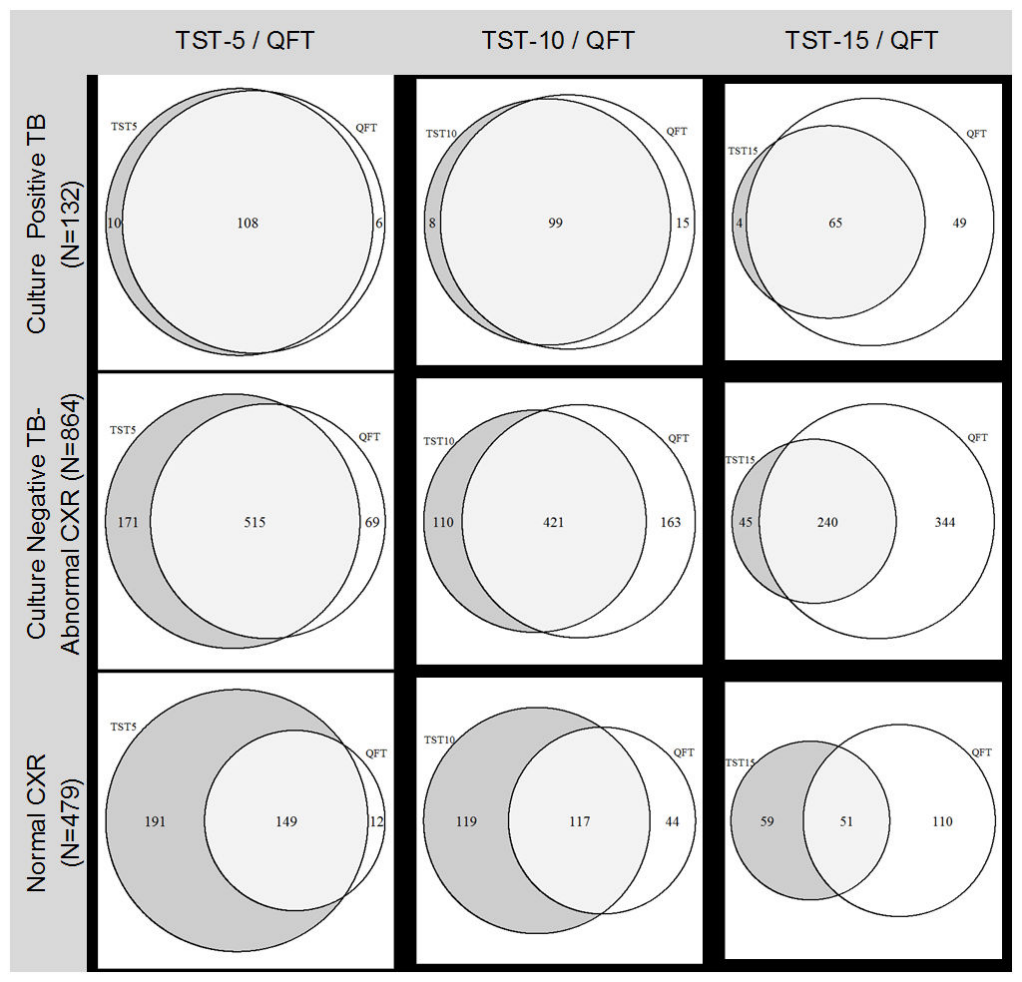
Mantoux tuberculin skin test (TST) and QFT-TB Gold In-Tube (QFT) response among participants, by tuberculosis group (rows) and three cutpoints for a positive TST (columns) at 5mm, 10mm, and 15mm induration. The area within a circle corresponds with the number of participants with a positive test; the area of the intersection of circles corresponds with the number of participants positive by both tests.

The sensitivity for detecting culture-confirmed tuberculosis was 86.4% (95% CI = 79.3%—91.7%) for QFT, 89.4% (82.8%—94.1%) for TST-5, 81.1% (73.3%—87.5%) for TST-10, and 52.3% (43.4%—61.0%) for TST-15 (Table 1). These results were significantly different for QFT versus TST-15 (Pearson's chi-squared probability [p]=<0.001) but not for QFT versus TST-5 (p=1) or TST-10 (p=0.12). Compared to those with TB, the prevalence of positive results for each test were much lower for applicants with Normal-CXR and intermediate for those with TB-CXR, some of whom may have had culture-negative tuberculosis, inactive tuberculosis or radiographic abnormalities not due to tuberculosis (Table 1). We also examined the agreement between the tests among the 132 applicants with culture-confirmed tuberculosis (TB) as shown in Figure 2. Both QFT and TST-5 were negative in 8 (6.1%), were both positive in 108 (81.8%), and 6 (4.5%) and 10 (7.6%) applicants were only positive by QFT or TST-5, respectively, reflecting the similar but imperfect sensitivity of QFT and TST for tuberculosis disease. Increasing the threshold for positive TST to 10 mm and 15 mm progressively decreased the number of subjects positive by TST, but decreased the sensitivity of TST compared to QFT. 

### Prevalence of LTBI for TST and QFT

To assess performance of the test for LTBI, we compared TST and QFT results among those without culture-confirmed tuberculosis. Compared with the TB group, discordance was similar but greater among the 864 participants in the TB-CXR group (Figure 2); 18.9% (n=163) with a TST <10 mm had a positive QFT, and 12.7% (n=110) of those with a TST >10 mm had a negative QFT. Among the 479 in the Normal-CXR group, the discordance was even greater between QFT and TST than observed for the two other groups; 9.2% (n=44) with a TST<10 mm had a positive QFT and 24.8% (n=119) of those with a TST >10 mm had a negative QFT. (A plot of QFT versus TST response, Figure S1, is available online). 

**Figure 3:**
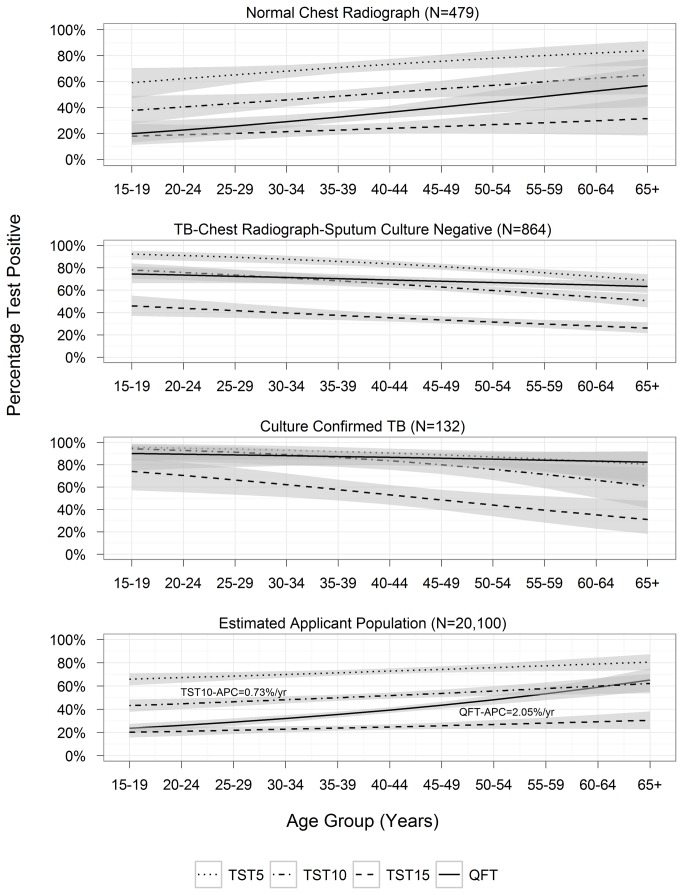
**Proportion positive QFT-TB Gold In-Tube (QFT) and Mantoux tuberculin skin test (TST) with positive at >5 mm induration (TST-5), >10 mm induration (TST-10), and >15 mm induration (TST-15) among four groups:** A) participants with chest radiographs not suggestive of TB (N=479); B) participants with chest radiographs suggestive of TB with negative sputum culture for TB (N=864); C) participants with chest radiographs suggestive of TB with positive sputum culture for TB (N=132); D) all U.S.-bound Vietnamese immigrant applicants (estimated). *APC=annual percent change per year of age.

The prevalence of a positive test was dependent on the participant’s age with notable differences by test method (Figure 3). For Normal-CXR (Figure 3), the percent test positive for QFT was just above 20% ages 15-19 years followed by an annual percent increase of 2.1% [0.7%—3.4%]. In contrast the prevalence of positive results were nearly 3-fold and 2-fold higher for TST-5 and TST-10, respectively with annual percent increases of 0.6 [0.0%—1.2%] for TST-5, 1.0% [0.1%—1.9%] for TST-10. Positive results for TST-15 among those 15-19 years of age were about 20% as with QFT but the annual percent increase was only 1.1% [0.7%—2.8%].  For TB-CXR (Figure 3), the percent test positive decreased with age, but less so for QFT (annual percent change was -0.3% [-0.6%—0.1%] for QFT, -0.4% [-0.6%—0.2%] for TST-5, -0.7% [-1.0%—0.36%] for TST-10, and -1.0% [-1.7%—0.4%] for TST-15.

When results from each group were weighted to simulate the test results in the applicant population (Figure 3D), we estimated a prevalence of 37.3% (34.7%—39.8%) for QFT; 72.0% (69.8%—74.3%), 50.8% (48.2%—53.3%), and 24.3% (22.1%—26.5%) for TST-5, TST-10, and TST-15. The estimated prevalence in the youngest age group of 15-19 years and annual percent change for QFT were 22% and 2.05% (1.29—2.81) per year, compared with 42% and 0.73% (0.07—1.40) per year for TST-10.

## Discussion

We evaluated the performance of the QFT assay and TST as criteria for the detection of pulmonary tuberculosis and LTBI in a population of 20 thousand adult Vietnamese visa applicants undergoing universal chest radiography followed by sputum cultures for those with any radiographic findings consistent with active or inactive pulmonary tuberculosis. Neither the TST at the most sensitive (5-mm) cutoff or QFT detected all the culture-positive pulmonary tuberculosis cases detected by the rigorous radiologic and microbiologic screening, detecting 89% and 86% with TST and QFT, respectively. These data suggest that QFT in this and similar high-risk populations is likely to perform as well as TST-5 when used as the initial test during two-stage screening in which a positive test for LTBI precedes chest radiography and sputum cultures is done for those with any radiographic findings consistent with tuberculosis.

In addition to similar sensitivity in detection of tuberculosis, two principal findings support the use of QFT over TST for two-stage tuberculosis screening in this BCG-vaccinated population. First, for detecting tuberculosis disease, we estimate positive test result for LTBI would lead to radiography of only 37% of the entire population with a positive QFT compared with 72% of those with a positive TST-5 with no difference in case detection. Second, for the goal of recommending treatment for those with a normal chest X-ray and the diagnosis of LTBI, the data in this study suggest that the specificity of QFT is superior to TST-10 Compared with the prevalence of a positive QFT at age 15-19 years of age, the 3-fold higher prevalence of a positive TST-5, and the 2-fold higher prevalence of TST-10, is most consistent with a TST reaction due to prior BCG vaccination or exposure to environmental bacteria, leading to an inappropriately high rate of LTBI. The increasing annual rate of QFT positivity (2.05% per year) is more consistent with the annual change prevalence of tuberculosis in the population (2.9%) than the increase for TST-10 positivity (0.75% per year). These findings indirectly support CDC’s recommendation[13] that IGRAs are the preferred test for persons likely to have received BCG vaccine. 

These findings have important implications for the use of IGRAs in tuberculosis control in the United States. In this population, when used instead of TST-5 or TST-10 in a two-stage tuberculosis screening process as a precursor to radiography and sputum collection, the use of QFT would detect a similar number of cases but require one-third fewer radiographs (one-half fewer in younger adults), and greatly reduce the number for whom LTBI therapy is recommended. Increasing the TST cutoff to 15 mm, as has been considered to reduce the false-positive rate for TST in some settings[31,32], would also result in fewer test positives but would greatly reduce (-52%) the sensitivity for tuberculosis disease and by inference the sensitivity for LTBI. 

For tuberculosis disease, the sensitivity of QFT (86%) and TST-10 (81%) was slightly higher in this study than was estimated in a 2007 meta-analysis (pooled sensitivity, QFT=67% [56%-78%], TST-10=72% [50%-95%])[33], although a 2012 meta-analysis estimated a very similar sensitivity of QFT (89% [87%-91%])[34]. Nonetheless, this study suggests that tuberculosis cases may be missed by two-stage tuberculosis screening with QFT or TST and this is why screening for TB disease in immigrant populations should also include, at a minimum, chest radiographs based on the results of symptom questionnaires and physical examination. Each year, approximately 500,000 immigrants are screened in the United States with IGRA or TST followed by chest radiograph[10], and 2.5 million newly arriving students, temporary workers, and other non-immigrants are recommended to undergo a similar two-stage screening. This strategy is generally effective, and has an added benefit of detecting latent tuberculosis infection, but may be missing approximately 10-20% of asymptomatic tuberculosis cases compared to the rigorously applied overseas radiography and collection of three sputum cultures from those with any radiographic findings suggestive of tuberculosis. 

The tuberculosis prevalence in this population was greater than in most published studies assessing QFT[35-37]. Compared with a 2010 national tuberculosis prevalence study in Vietnam[38], the high tuberculosis prevalence (1.0%) we report may be attributed to performing three serial sputum cultures instead of one. Our findings are similar to the 1.3% reported in a previous study of applicants in Vietnam, when culture was demonstrated to be approximately 3 times as sensitive as smears[22]. 

This study has several limitations. One, no acid-fast bacilli sputum smears or cultures were obtained for applicants with chest radiographs not suggestive of tuberculosis. It is unlikely, but possible, that these persons had tuberculosis disease. Two, except for those with tuberculosis disease, the tuberculosis infection status cannot be determined with certainty because there is no gold standard for LTBI detection, and therefore the specificity could not be calculated. Third, the BCG immunization history for applicants was not obtained; we assumed that all those enrolled had been immunized with BCG at birth. Fourth, our study included only one applicant with HIV; therefore, our conclusions for tuberculosis screening should be limited to HIV-negative persons. (CDC requires all HIV-positive immigrant applicants to have sputum testing for *M. tuberculosis*.) 

Despite these limitations, this study indicates that QFT is superior to TST in BCG vaccinated populations since it provides equal sensitivity in the commonly-used two-stage screening process for tuberculosis disease and yields fewer positives than TST at 10 mm (only half among adults 15-19 years) suggesting superior specificity for LTBI. 

## Supporting Information

Figure S1 **Mantoux tuberculin skin test (TST) and QFT-TB Gold In-Tube (QFT) response among three groups: participants with chest radiograph not suggestive of TB (N=479); participants with chest radiograph suggestive of TB but negative sputum cultures (N=864); participants with chest radiograph suggestive of TB with positive sputum cultures for TB (N=132).** QFT response considered positive when > 0.35 IU/mL (horizontal reference line). Shaded area corresponds to TST induration <5 mm (light gray); 5-10 mm (medium gray); 10-15 mm (medium-dark gray); 15 mm or greater (dark gray). Logarithmic transformation of QFT (note: reference line at cutoff for positive QFT log (0.35)) and jittering of points were done to improve visual display.
(PNG)
