# Supplementary figures and images for: Tuberculosis Screening by Tuberculosis Skin Test or QuantiFERON®-TB Gold In-Tube Assay among an Immigrant Population with a High Prevalence of Tuberculosis and BCG Vaccination
Source: PLoS One. 2013 Dec 19;8(12):e82727. doi: 10.1371/journal.pone.0082727 (PMC3868593; doi:10.1371/journal.pone.0082727)

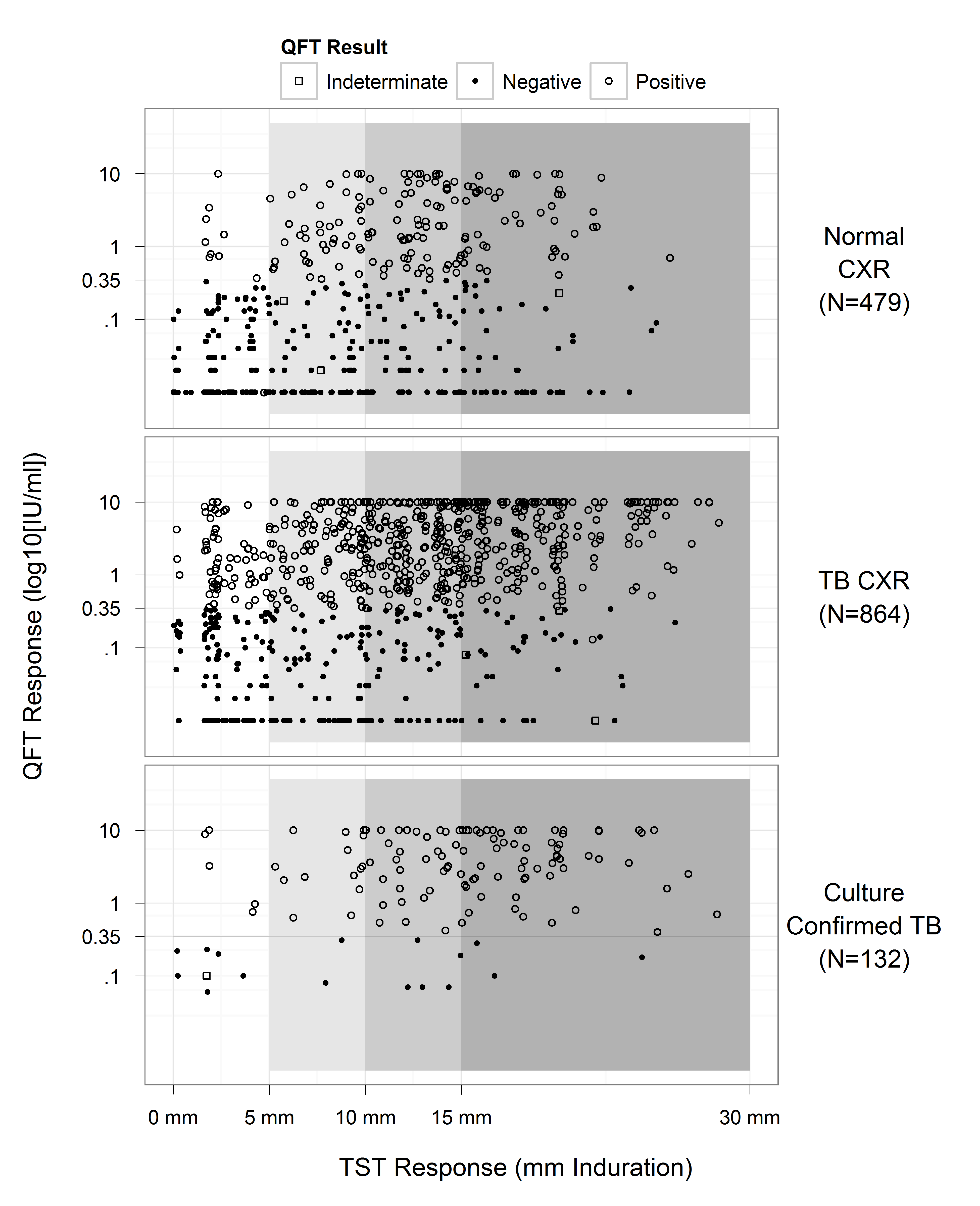

Supplement: Figure S1 — Mantoux tuberculin skin test (TST) and QFT-TB Gold In-Tube (QFT) response among three groups: participants with chest radiograph not suggestive of TB (N=479); participants with chest radiograph suggestive of TB but negative sputum cultures (N=864); participants with chest radiograph suggestive of TB with positive sputum cultures for TB (N=132). QFT response considered positive when > 0.35 IU/mL (horizontal reference line). Shaded area corresponds to TST induration <5 mm (light gray); 5-10 mm (medium gray); 10-15 mm (medium-dark gray); 15 mm or greater (dark gray). Logarithmic transformation of QFT (note: reference line at cutoff for positive QFT log (0.35)) and jittering of points were done to improve visual display. (PNG) [file pone.0082727.s001.png]
